# Dynamics of Virological and Clinical Response Parameters of Bulevirtide Treatment for Hepatitis D: Real-World Data

**DOI:** 10.1016/j.gastha.2024.01.001

**Published:** 2024-01-05

**Authors:** Alexander Killer, Smaranda Gliga, Carolin Lohr, Christian Weigel, Björn-Erik Ole Jensen, Nadine Lübke, Andreas Walker, Jörg Timm, Johannes Bode, Tom Luedde, Hans H. Bock

**Affiliations:** 1 Department for Gastroenterology, Hepatology and Infectious Diseases, Medical Faculty and University Hospital Düsseldorf, Heinrich Heine University Düsseldorf, Düsseldorf, Germany; 2 Institute of Virology, Medical Faculty and University Hospital Düsseldorf, Heinrich Heine University Düsseldorf, Düsseldorf, Germany

**Keywords:** Antiviral-Treatment, Entry-Inhibitor, Delta, Treatment Kinetics

## Abstract

**Background and Aims:**

The entry inhibitor bulevirtide represents the first specific treatment for hepatitis-D virus (HDV)-infected patients. In clinical trials, around 80% of patients achieve normalization of alanine aminotransferase (ALT) with about 60% virological response after 1 year, but little is known about the dynamics of responses and clinical predictors of treatment outcomes. We report our single-center data from 15 patients and describe response dynamics, clinical outcomes, and predictive factors for treatment response.

**Methods:**

Retrospective data from 15 patients have been analyzed at our department who started treatment with bulevirtide between 10/2020 and 08/2022. According to our standard procedures, laboratory parameters were controlled monthly; transient elastography was performed every 3 months, and the treatment duration was 12 months.

**Results:**

Treatment response rates after 1 year of treatment were similar to published data from clinical trials. ALT normalization usually occurs between months 2–6 of treatment, followed by a virological response after ≥6 months. Patients with more severe hepatitis at the start of treatment were less likely to respond in the first year of treatment. Loss of HDV-RNA was observed in one-third of patients after ≥1 year of treatment. Low body mass index and high alpha-fetoprotein at baseline were possible predictors of a delayed treatment response.

**Conclusion:**

Bulevirtide is a safe treatment option for HDV, leading to a fast hepatological response. Of note, decrease in transaminases precedes virological response. Patients with high viral load and ALT levels respond slower, but nonresponders (as classified by Food and Drug Administration criteria) still show a reduction in viremia. Longer observation periods are required to determine the optimal duration of bulevirtide monotherapy.

## Introduction

Hepatitis B/D coinfection is the most severe form of viral hepatitis.^1^ Hepatitis D virus (HDV), a defective RNA virus that requires hepatitis-B-surface-antigen (HBsAg) to complete its virion production, relies on hepatitis B virus (HBV) coinfection, either as a concurrent coinfection or as a superinfection of HBV-infected patients.^2^^,^^3^ Approximately 250 million people worldwide are living with HBV infection. Estimates for HDV vary from 5% to 15% of HBV-infected patients, corresponding to 12.5–37.5 million people worldwide. Underdiagnosis is discussed, particularly in high-prevalence regions in South America, West Africa, and Central Asia.^4^^,^^5^ Prevalence in Western countries is lower, but it is also influenced by recent migration. Hence, there is a need for a growing awareness of HDV infection among clinical practitioners. As of September 2020, bulevirtide is available as the first conditionally approved treatment option for HBV/HDV-coinfected patients in Europe^6^ with full marketing authorization being granted in April 2023 by the European Medicines Agency. Bulevirtide is a novel entry inhibitor that targets the sodium-taurocholate-co-transporting polypeptide receptor and thus the entry of HDV and HBV virions into hepatocytes.^7^, ^8^, ^9^ The criteria for treatment success in HDV^10^ used in the MYR 202 and 301 clinical trials were assessed by virological, or a combined biochemical and virological response: Bulevirtide at a dose of 2 mg per day achieved a ≥2-log level reduction of or undetectable HDV-RNA levels in 54% after 24 weeks and a combined response of undetectable HDV-RNA or a decrease of ≥2 log10 IU per mL with normalization of alanine aminotransferase (ALT) in 45% after 48 weeks.^11^^,^^12^ Given its stand-alone status and good treatment tolerance even in patients with compensated cirrhosis, this represents a step change in the treatment of HDV-coinfected individuals.

We report our real-world single-center data from 15 patients treated with bulevirtide for 1 year and evaluate the dynamics of treatment response, particularly monthly changes of biochemical and virologic parameters and the effect of treatment on liver stiffness. It was also investigated whether clinical factors could be identified to predict response to therapy, as approximately 20% of patients did not meet currently accepted endpoint criteria for treatment.

## Patients and Methods

In patients diagnosed with HBV/HDV co-infection, the indication for HDV-treatment initiation with bulevirtide was based on biochemical and clinical criteria (hepatitis and/or evidence of liver fibrosis or compensated cirrhosis). Patients signed an informed consent form (#5350 approval by the ethics committee of the Medical Faculty of the HHU Düsseldorf). At the start of treatment, each patient was informed about bulevirtide preparation and trained for self-application. After treatment initiation, patients were tested monthly for:

HBV-DNA, HBsAg, HDV-RNA, ALT, aspartate aminotransferase (AST), white-blood-cell count, platelets, alpha-fetoprotein (AFP), and bile acids. HDV genotype was determined once.

The diagnosis of cirrhosis was based on histology, if available, ultrasound, elastography, and/or portal hypertension (varices, splenomegaly, and thrombocytopenia).

### Laboratory Analysis

HBV and HDV parameters were analyzed as part of virological routine diagnostics. HBV-serology was done on the ARCHITECT i2000SR (Abbott), and anti-HDV was determined on a Liaison-XL (DiaSorin). HBV-DNA was quantified with the Cobas HBV test (07000979190) on a Cobas 6800 (both Roche).

### Extraction of HDV-RNA and RT-PCR

Viral RNA from 400 μL of plasma was extracted automatically using the EZ1 Virus Mini Kit v2.0 on an EZ1 Advanced XL robot or manually with the QIAamp Viral RNA Mini Kit (both Qiagen) according to the manufacturer’s protocol. RNA was eluted in a volume of 60 μL and stored at −80 °C. RT-qPCR was performed with the RealStar HDV RT-PCR Kit 1.0 (Altona Diagnostics, #401003). To reduce hands-on time and avoid repeat freeze-thaw cycles, PCR mixes were prepared in batches, frozen in aliquots, and stored at −20 °C until usage. Before amplification, secondary HDV-RNA structures were melted at 95 °C for 3 minutes, and denatured viral RNA was added to the thawed PCR mixes. Reverse transcription conditions were 20 minutes at 55 °C followed by 2 minutes at 95 °C denaturation, and then 40 qPCR cycles, each 15 s at 95 °C, 45 s annealing at 55 °C and 15 s extension at 72 °C.

### HDV-Genotyping

HDV genotype was determined by sequence analysis. Amplification and sequencing were done as previously described.^13^ In brief, RNA was reverse transcribed in vitro with Superscript III (SSIII, Invitrogen, #18080085) and primer HDV-771R (CGGTCCCCTCGGAATGTTG) with the previously described conditions: 10 minutes at 25 °C, 60 minutes at 42 °C, 30 minutes at 50 °C, 30 minutes 55 °C, 15 minutes at 75 °C and 4 °C.^14^, ^15^ A 2-step semi-nested PCR was performed with GoTaq HotStart-Polymerase (Promega, #M7401) according to the manufacturer’s protocol and the following primer combinations for PCR I:

HDV-891F (AGGTCGGACCGCGAGGAGGT);

HDV-339R (GCTGAAGGGGTCCTCTGGAGGTG) and PCR II:

HDV-912F (GAGATGCCATGCCGACCCGAAGAG);

HDV-339R (GCTGAAGGGGTCCTCTGGAGGTG).

PCR products were sent for sequencing (SeqIT, Kaiserlautern, Germany). Illumina data were processed with SeqIT’s internal bioinformatics pipeline.^16^ Briefly, sequence data were mapped to an HDV reference sequence (GenBank M21012), primer sequences were deleted, and a consensus sequence was generated. The consensus sequence was used as a new reference, and this process was repeated up to 4 times. The final consensus sequence was used for genotyping and subsequent analyses.

### Elastography

Shear wave elastography was performed at least every 3 months to assess liver stiffness using the Canon Aplio i800. Results are presented in kPa.^17^^,^^18^

### Statistics

Normally distributed quantitative values were compared by t-test, and variables with a non-normal distribution were compared by the Mann-Whitney U-test. Categorical parameters were analyzed by Fisher’s exact test. Prism Graph Pad and Windows Excel were used for statistical analysis and graphs. The Wilcoxon signed rank test and repeated measures ANOVA were used to compare changes during treatment. The significance level was set at 0.05.

## Results

### Cohort

A total of 17 patients were treated. One patient moved to another city 3 months after treatment and was therefore excluded from the analysis. One patient died during treatment from hepatocellular carcinoma recurrence followed by immunotherapy and was therefore also excluded.

The median age was 41 years, and sexes were almost evenly distributed (Table). One patient had been treated for only 10 months by the time of analysis. Almost half of the patients had CHILD A cirrhosis, and the median liver stiffness was 10.6 kPa. One patient had symptoms of decompensated cirrhosis in the past but had compensated cirrhosis (CHILD-Pugh A) at the start of treatment. All patients were HBV-DNA negative at start of bulevirtide treatment, and all patients under continuous nucleoside/nucleotide analog (NA) treatment remained HBV-DNA negative. Three out of 15 patients did not receive NA treatment at the start of bulevirtide treatment. One of these patients developed an HBV reactivation after 5 months of bulevirtide (HBV-DNA 85 IU/mL), reaching 25,800 IU/mL at month 7. NA treatment was therefore initiated, and HBV DNA was undetectable 1 month later. In a second patient with liver cirrhosis, whose HBV-DNA levels were repeatedly undetectable before initiation of bulevirtide treatment, NA treatment was initiated at month 6 because low HBV-DNA levels (<30 IU/mL) became detectable. One patient without NA treatment remained HBV DNA negative throughout the observation period. Most of the patients (73%) had received prior treatment with interferon-α. The median HDV viremia at treatment start was 497,000 IU/mL, and all patients had HDV genotype 1 (Table).

**Table:** Baseline Patient Characteristics of 15 Patients Treated With Bulevirtide for 1 Year Included in the Retrospective Analysis

Variable	n (%)	Median	Mean	Standard deviation
Age [y]		41	42	12
BMI [kg/m^2^]		27	26	4
ALT [U/L]		78	137	119
AST [U/L]		58	81	47
Female	7 (47)			
Liver cirrhosis CHILD A	7 (47)			
Prior IFNα treatment	11 (73)			
NA treatment at baseline	12 (80)			
HDV-RNA [IU/mL]		497,000	3,324,000	7,911,000
HBsAg [U/mL]		22,600	18,300	12,600
HBeAg positive	2 (13)			
HDV gentotype 1	15 (100)			
Liver stiffness [kPa]		10.6	13	6.8
White blood cells [/μmL]		4200	4800	1900
Platelets [1000/μL]		166	164	66
Bile acids [μmol/L]		6.9	11.7	11.8
AFP [μg/L]		5.4	7.4	5.8

BMI, body mass index; HBeAg, hepatitis B e antigen; IFNα, interferon-α.

### Virological Response

**Figure 1:**
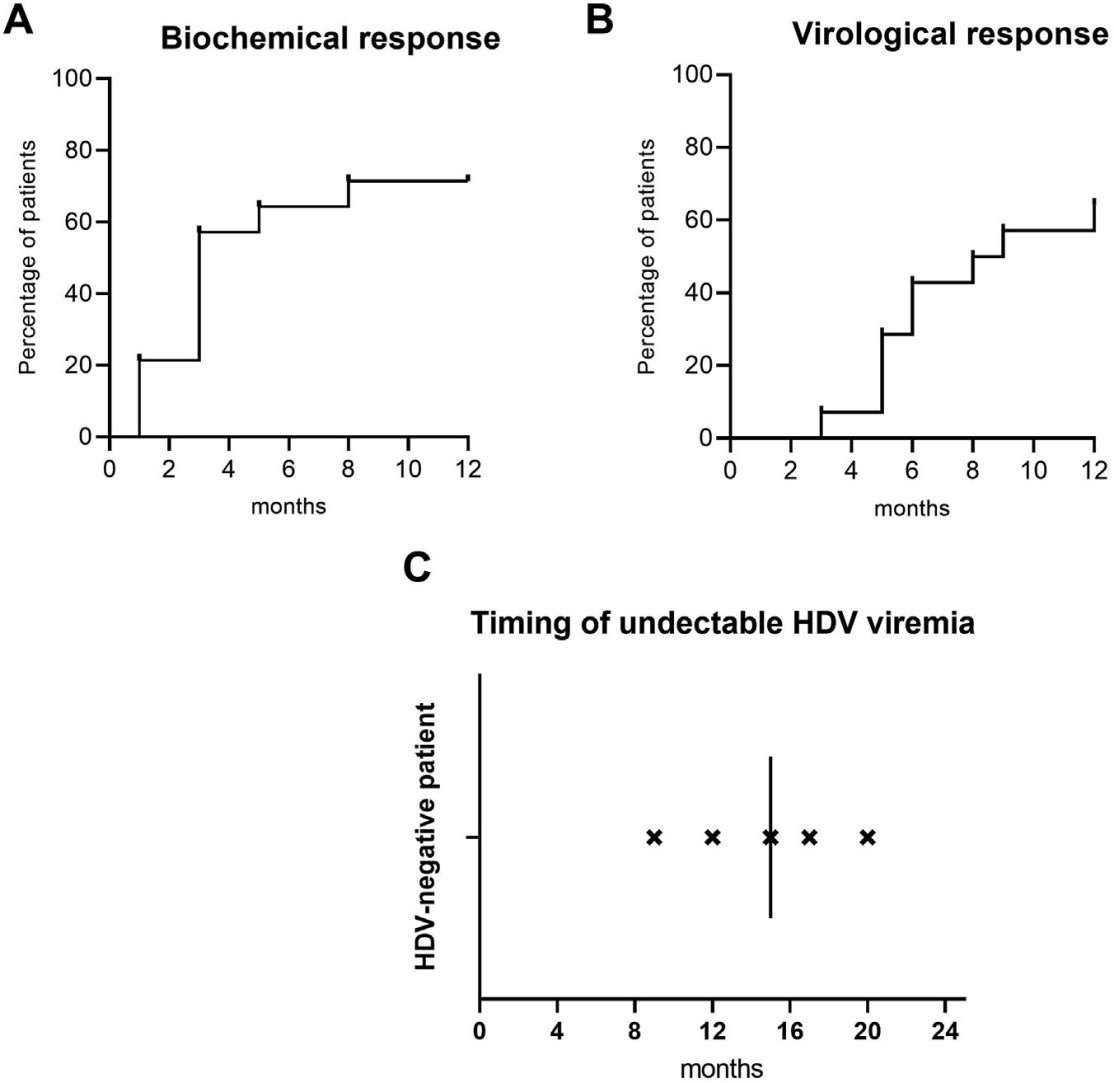
Dynamics of treatment response: (A) First treatment responses occurred after 1 month for ALT normalization and (B) after 3 months for HDV-decrease of ≥2 log. In general, ALT response is seen more frequently and earlier than virological response. (C) HDV-RNA became undetectable after the first year of treatment in 3/5 patients. Bar: median time to undetectable HDV-RNA levels.

**Figure 2:**
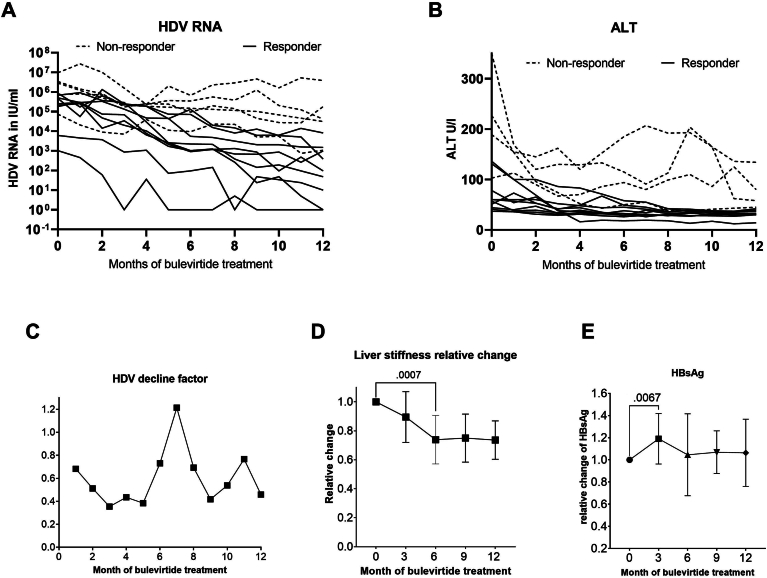
Individual treatment responses for (A) HDV-RNA and (B) ALT. Nonresponders for both parameters have higher baseline values in HDV-viremia and ALT elevation but still show somewhat reduced HDV and ALT levels. ALT decline occurs earlier during treatment than HDV-RNA decline. (C) HDV-viremia is reduced to 60% on average, but initial treatment response is faster with slower to no response from months 6–8 and then again accelerated as of month 9. (D) Liver stiffness, presented as relative changes of median values, decreases during the first 6 months to 73% of baseline values and then stabilizes, which may reflect a predominant effect on hepatic inflammation during the first months of treatment. (E) Relative changes of HBsAg.

Six out of 15 patients (43%) had a virologic response defined as a decline in HDV-RNA levels of at least 2 log at 6 months and 9/14 at 12 months (64%) (Figure 1A). Median HDV levels had declined significantly by 3 months of treatment (*P* ≤ .001) and continued to decrease at 12 months (*P* ≤ .001). The earliest virologic response to treatment was observed at 3 months (Figure 1B), the latest at 12 months, and the median treatment duration to virologic response was 6 months. The median monthly HDV-viremia reduction factor for all patients was 0.59 for all months of treatment, indicating that HDV-RNA decreased to ∼60% of the previous month (Figure 2C). The decrease was faster in the first months, with a slower kinetic from months 6–9 and then again a faster decline. Patients with HDV RNA >500,000 IU/mL at baseline were less likely to respond to HDV treatment by 12 months than patients with lower HDV viremia (*P* = .088) (Figure 2A).

Only 2 patients had undetectable HDV-RNA after the first year of treatment (Figure 1C). At the time of analysis, 5 patients had undetectable HDV viremia. Treatment duration until HDV-RNA was undetectable was at least 9 months (median: 15 months); the latest response so far occurred 20 months after treatment onset (Figure 1C).

Median HBsAg levels did not change with bulevirtide treatment at 12 months, and there were no patients with HBsAg loss. There was a trend toward a moderate increase in HBsAg levels between start and 3 months of treatment (*P* = .0549), which wore off over the ongoing duration of therapy (Figure 2E). HBV-DNA was undetectable in serum in all patients at month 12.

### Biochemical Response

**Figure 3:**
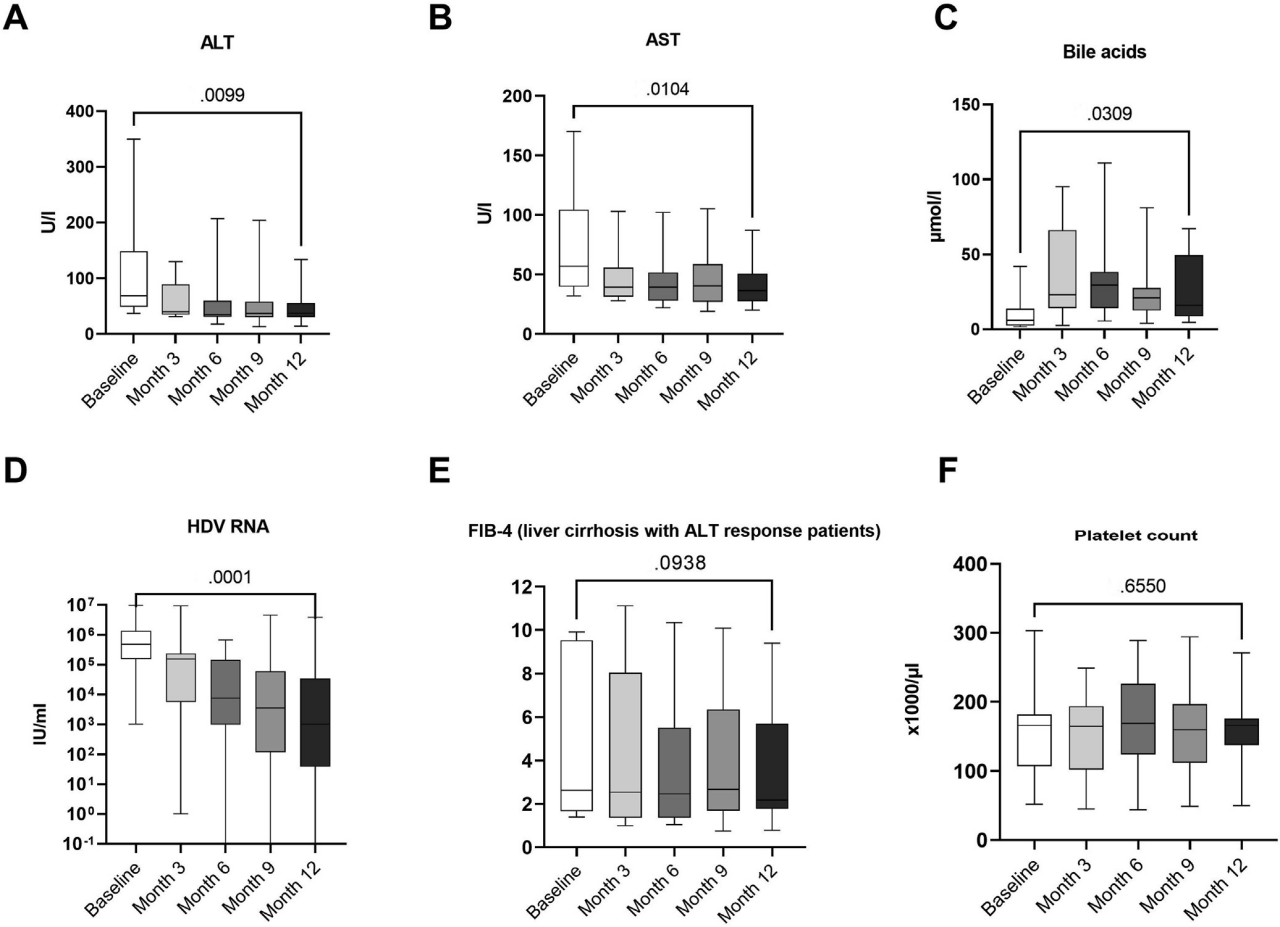
Change of mean values over first year of bulevirtide treatment. Analysis with ANOVA showed significant changes for (A) ALT, (B) AST, (C) bile acids, and (D) HDV RNA. (E) Changes in FIB-4 in patients with hepatological response and liver cirrhosis were not significant, but with a slight trend toward a reduction in FIB4. (F) Platelet count stayed at similar over treatment.

**Figure 4:**
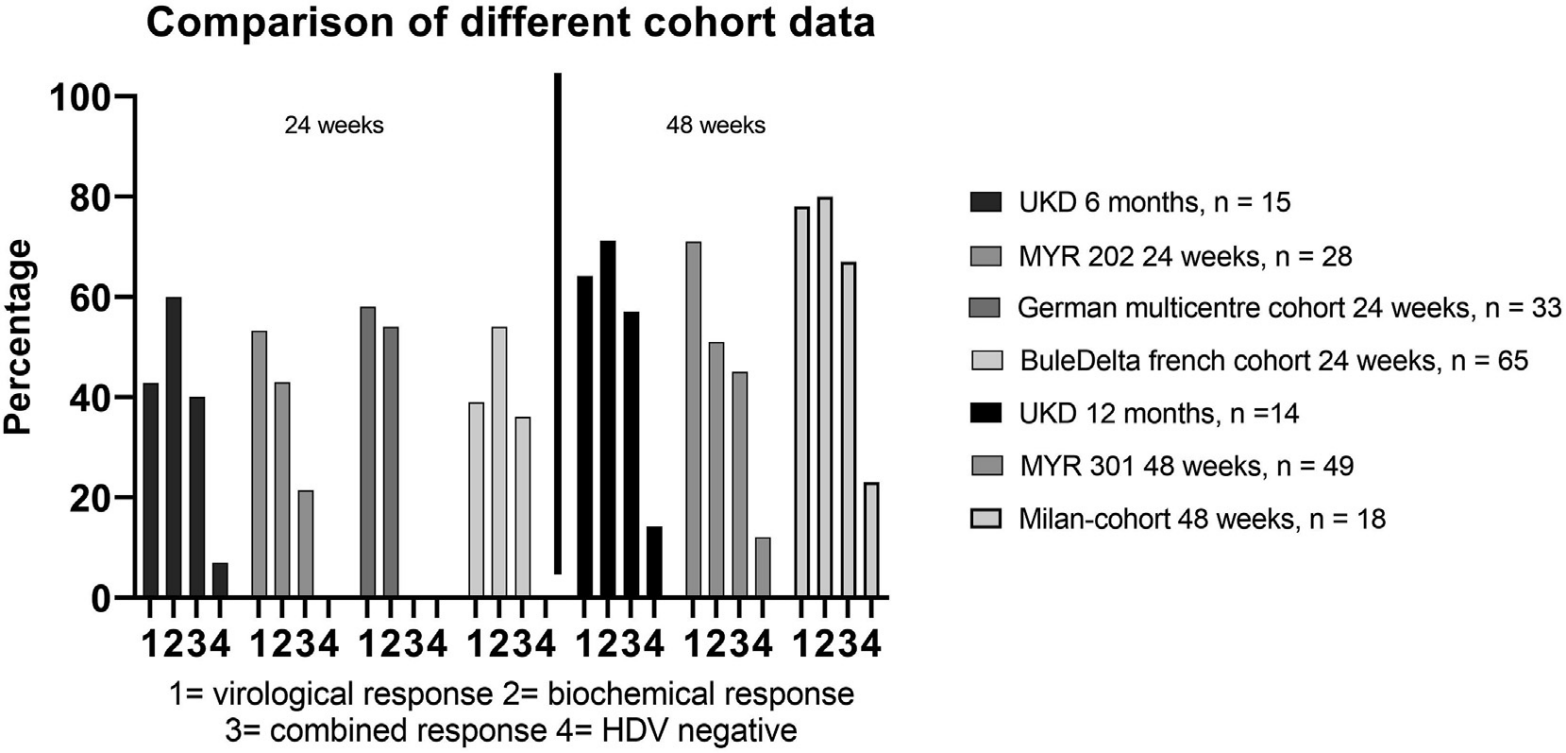
Data from this report (=UKD) compared with available data from phase 3 studies MYR 202 and MYR 301^11^^,^^12^ and various real-world data.^19^, ^20^, ^21^ Outcomes are similar, with the highest treatment response rates reported for the Milan liver cirrhosis cohort.

Nine out of 15 (60%) patients had a biochemical response (normalization of ALT) after 6 months and 10/14 (71%) after 12 months of treatment (Figure 1B). The change in median ALT levels of all patients from baseline to month 3 did not reach statistical significance, whereas changes after months 6 and 12 were significant (Figure 3 changes of biochemical markers during treatment, ALT changes Figure 3A). The earliest ALT normalization occurred after 1 month of treatment, and the median time to response was 3 months (Figure 1A). Six out of 15 (40%) patients had a combined biochemical and virological response after 6 months and 8/14 (57%) after 12 months (Figure 4). Two patients had normal ALT levels but no virological response, and 1 patient had a virological but no biochemical response at month 12. Median platelet counts did not change (Figure 3F), even when considering the subgroup of patients with positive treatment responses individually, which suggests no measurable effect on portal hypertension within the observation period of 1 year.

### Side Effects

No severe side effects occurred with the treatment. No local reactions after injection or pruritus were reported, and no patient interrupted treatment. One patient experienced an HBV reactivation, which quickly responded to the start of NA treatment (see above).

### Liver Stiffness

Median liver stiffness declined from baseline median 10.6 kPa**–**9.2 kPa at month 3, 7.8 kPa at month 6 and remained stable at months 9 (7.3 kPa) and 12 (7.6 kPa). Changes compared to baseline were significant (*P* ≤ .001).

### Predictors for Treatment Response

**Figure 5:**
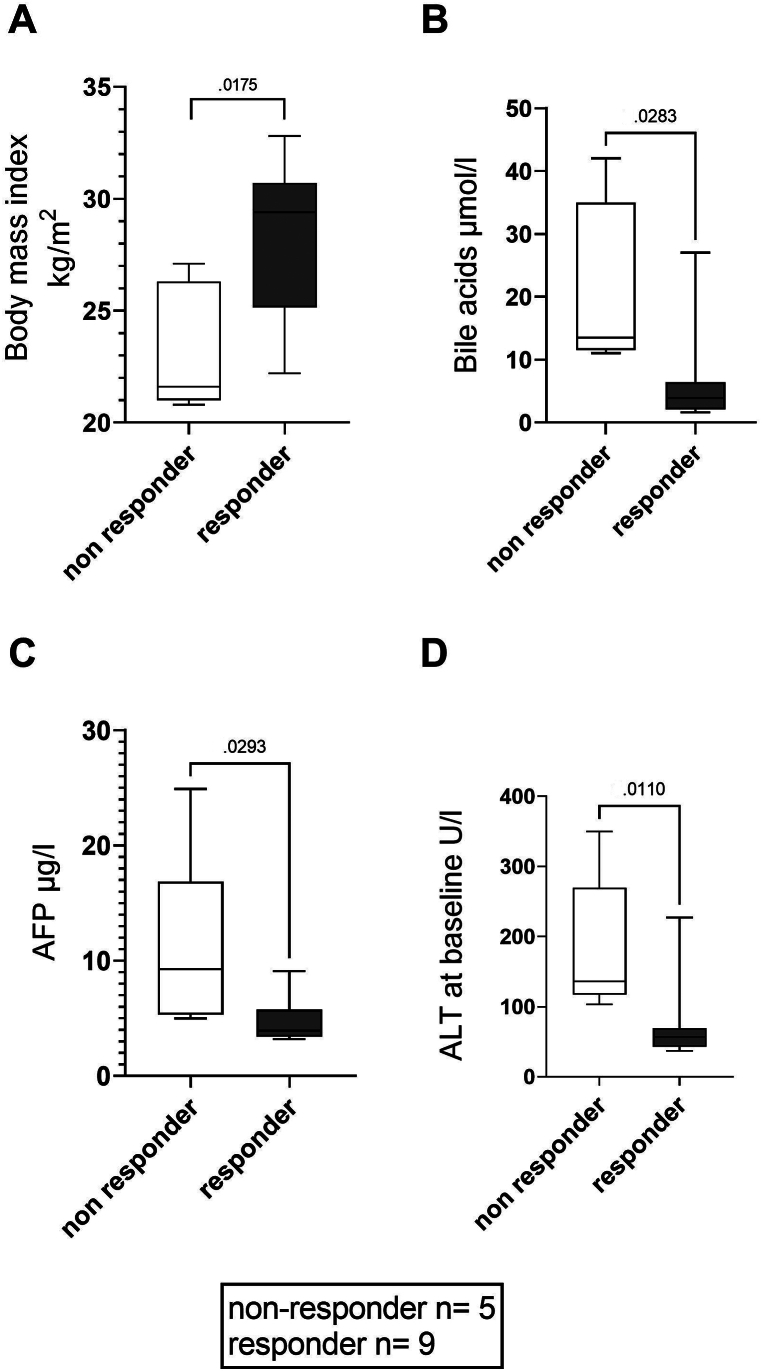
Comparison of baseline values of virological nonresponders to responders: We found higher BMI, lower levels of AFP, lower baseline bile acid, and lower ALT levels to be associated with treatment response. Box plot graphs show mean values and minimum-maximum. BMI, body mass index.

To determine possible predictive factors for differential response to treatment, we performed a Fisher exact test for sex, liver cirrhosis, low platelet count (=below 150,000/μL), low white blood cell count (below 4000/μL), high baseline HDV viremia (>500,000 IU/mL), and high level of liver inflammation (ALT >100 U/L). Sex, liver cirrhosis, platelet count, and white blood cell count were found to be independent of treatment response. High ALT levels at baseline were associated with lower hepatologic and virologic responses to treatment (Figure 5), which was confirmed by Mann-Whitney U-test comparing baseline ALT levels for virological responders and nonresponders (*P* = .011). Patients with virological nonresponse had lower body mass index (*P* = .017, Figure 5A), higher baseline bile acids (*P* = .028, Figure 5B) and higher AFP-levels at baseline (*P* = .029, Figure 5C). There was also a trend toward lower treatment responses in patients with high HDV-viremia levels at baseline (*P* = .059). Of 2 hepatitis B e antigen (HBeAg)-positive patients, one had a combined response and the other a biochemical response.

## Discussion

We report real-world, retrospective bulevirtide treatment data for 15 patients, with treatment response rates after 12 months of treatment for 14 patients. Hepatic and virologic response rates for the 24-week and 1-year treatment periods were comparable to data from the MYR202 and MYR301 clinical trials and previously published real-world data.^11^^,^^12^ Thus, expected treatment response rates are 60%–70% for virologic response and 70%–80% for ALT normalization after 1 year of therapy. With regard to clinically relevant outcome criteria such as survival, development of cirrhosis, decompensation, liver transplantation, and improvements in quality of life, it is important to consider whether these virological and biochemical outcomes are predictive. In particular, the small group of patients with ALT normalization but without virological response is interesting in this context. It is unclear whether these patients actually have worse outcomes in terms of overall success than patients with a combined response, especially since these patients experienced a decline of more than 1 log (defined as ‘intermediate virologic response’ by Dietz-Fricke et al^19^). Studies of HBV responses to NA treatment have shown that early ALT normalization is key to preventing severe hepatologic events.^22^ Therefore, it does not seem appropriate to categorize patients with biochemical responses as “treatment nonresponders”. The reduction in HDV-RNA per month averaged a factor of 0.6. Zöllner et al^23^ reported a similar reduction rate of 0.63, which is consistent with the data presented here, suggesting a relatively slow virological response to treatment with bulevirtide.

Of note, normalization of ALT under bulevirtide treatment occurs earlier than the decline of HDV-RNA levels, which contrasts with the response seen to NA treatment in hepatitis B. At first sight, this observation seems somewhat counterintuitive, but it may reflect the mechanism of action of bulevirtide. Inhibition of viral entry prevents infection of new hepatocytes, resulting in reduced inflammation, but does not prevent the release of infectious HDV particles from already infected cells. This could also explain why high HDV-viremia and high ALT levels at baseline are negative predictors of early response to treatment, since in these patients the percentage of already infected hepatocytes is high and entry inhibition takes longer to reduce HDV-production. In many of our patients, we noted a decreased rate of HDV clearance after about 6 months, which accelerated again after about 9 months. This virological dynamic, if confirmed in larger cohorts, needs to be further evaluated.

Loss of HDV-RNA was achieved in one-third of patients but mostly occurred after 1 year or more of treatment (Figure 1C), showing the possible long-term effects of treatment. Whether an undetectable HDV-viremia correlates with a cure for HDV-coinfection remains unclear. Anolli et al^24^ reported a case of HDV cure after 3 years of treatment in a patient with liver cirrhosis and no signs of HDVAg expression in liver biopsy. Since late responses to bulevirtide treatment are observed, interruption of treatment in patients who do not reach a combined response (2 log HDV decline, ALT normalization) needs to be carefully evaluated, especially if ‘intermediate’ virological or biochemical responses are observed. Nonetheless, the significant portion of nonresponders after 1 year of treatment underlines the need for novel treatment options, alone or in combination with bulevirtide, and stopping rules for bulevirtide.

The only treatment response that would unambiguously justify a treatment stop of bulevirtide is a confirmed HBsAg loss.^25^ In line with clinical studies and published real-world data, we did not observe changes in quantitative HBsAg levels after 24 weeks and 1 year of treatment (Figure 3F). During the initiation of treatment, however, we observed a slight but noticeable increase of HBsAg, which faded off thereafter. This slight increase in HBsAg was also seen in real-life data from 2023 from 114 patients, of whom 20 had repeated HBsAg measurements.^19^ This temporary HBsAg increase might reflect a temporary dampening of immune responses since liver inflammation, as mirrored by ALT levels, decreases. The mechanism could be similar to HBV reactivation following HCV treatment.^26^ In line with this, we observed a mild HBV-flare in a NA-naïve patient 3 months after treatment started, necessitating NA-start.

We report a significant decrease in liver stiffness during bulevirtide treatment in our patients (Figure 2D). Because changes occurred within the first 6 months of treatment, which leveled off until month 12, this most likely reflects reduced liver inflammation. Longer observational studies are required to evaluate whether this translates into reduced fibrosis. Regarding possible predictive factors of treatment response, we found that patients with baseline ALT >100 U/L, higher baseline bile acid concentrations, lower body mass index, and higher AFP levels had fewer treatment responses after 1 year. This has to be confirmed in larger cohorts. Higher concentrations of bile acids at baseline were already described as a negative predictive factor by Deterding et al.^27^ A question for further evaluation is if those late or nonresponders all achieve a response after longer treatment duration and if they have a higher rate of clinical adverse effects, such as development of cirrhosis, hepatic decompensation, or hepatocellular carcinoma. The power of the present analysis is limited because of its retrospective nature and the small number of patients. In addition, 2 patients who started treatment but could not be followed up were not included in the retrospective analysis.

However, treatment at a single center allowed for coherent data collection. In addition, performing the measurements in a centralized diagnostic virology laboratory allowed the determination of absolute HDV-RNA levels at monthly intervals. This circumstance is relevant since consistent handling of testing by 1 laboratory is important for reliable quantitative HDV viral loads,^28^ which is met for the retrospective real-world data summarized here.

In conclusion, bulevirtide is a safe treatment option. No serious adverse events occurred during the observation period, and no patient had to discontinue treatment due to adverse events. The data summarized here suggest that the majority of patients appear to benefit from treatment and that HDV RNA loss can be achieved in one third after ≥1 year.
